# Recommendations from the Italian intersociety consensus on Perioperative Anesthesa Care in Thoracic surgery (PACTS) part 2: intraoperative and postoperative care

**DOI:** 10.1186/s13741-020-00159-z

**Published:** 2020-10-23

**Authors:** Federico Piccioni, Andrea Droghetti, Alessandro Bertani, Cecilia Coccia, Antonio Corcione, Angelo Guido Corsico, Roberto Crisci, Carlo Curcio, Carlo Del Naja, Paolo Feltracco, Diego Fontana, Alessandro Gonfiotti, Camillo Lopez, Domenico Massullo, Mario Nosotti, Riccardo Ragazzi, Marco Rispoli, Stefano Romagnoli, Raffaele Scala, Luigia Scudeller, Marco Taurchini, Silvia Tognella, Marzia Umari, Franco Valenza, Flavia Petrini, on behalf of AIPO, Associazione Italiana Pneumologi Ospedalieri; SIAARTI, Società Italiana di Anestesia Analgesia Rianimazione Terapia Intensiva; SIC, Società Italiana di Chirurgia; SICT, Società Italiana di Chirurgia Toracica; SIET, Società Italiana di Endoscopia Toracica; SIP, Società Italiana di Pneumologia

**Affiliations:** 1 Department of Critical and Supportive Care, Fondazione IRCCS Istituto Nazionale dei Tumori, Milan, Italy; 2 Division of Thoracic Surgery, ASST Mantova, Mantova, Italy; 3 Division of Thoracic Surgery and Lung Transplantation, Department for the Treatment and Study of Cardiothoracic Diseases and Cardiothoracic Transplantation, IRCCS ISMETT - UPMC, Palermo, Italy; 4 Department of Anesthesia and Critical Care Medicine, National Cancer Institute “Regina Elena”-IRCCS, Rome, Italy; 5 Department of Critical Care Area Monaldi Hospital, Ospedali dei Colli, Naples, Italy; 6 Division of Respiratory Diseases, IRCCS Policlinico San Matteo Foundation and Department of Internal Medicine and Therapeutics, University of Pavia, Pavia, Italy; 7 Department of Thoracic Surgery, University of L’Aquila, L’Aquila, Italy; 8 Thoracic Surgery, AORN dei Colli Vincenzo Monaldi Hospital, Naples, Italy; 9 Department of Thoracic Surgery, IRCCS Casa Sollievo della Sofferenza Hospital, San Giovanni Rotondo, FG Italy; 10 Department of Medicine, Anaesthesia and Intensive Care, University Hospital of Padova, Padova, Italy; 11 Thoracic Surgery Unit - San Giovanni Bosco Hospital, Turin, Italy; 12 Thoracic Surgery Unit, University Hospital Careggi, Florence, Italy; 13 Thoracic Surgery Unit, ‘V Fazzi’ Hospital, Lecce, Italy; 14 Anesthesiology and Intensive Care Unit, Azienda Ospedaliero Universitaria S. Andrea, Rome, Italy; 15 Thoracic Surgery and Lung Transplant Unit, Fondazione IRCCS Ca’ Granda Ospedale Maggiore Policlinico, Milan, Italy; 16 Department of Morphology, Surgery and Experimental Medicine, Azienda Ospedaliero-Universitaria Sant’Anna, Ferrara, Italy; 17 Anesthesia and Intensive Care, AORN dei Colli Vincenzo Monaldi Hospital, Naples, Italy; 18 Department of Health Science, Section of Anesthesia and Critical Care, University of Florence, Florence, Italy; 19 Department of Anesthesia and Critical Care, Careggi University Hospital, Florence, Italy; 20 Pneumology and Respiratory Intensive Care Unit, San Donato Hospital, Arezzo, Italy; 21 Clinical Epidemiology Unit, Scientific Direction, Fondazione IRCCS San Matteo, Pavia, Italy; 22 Respiratory Unit, Orlandi General Hospital, Bussolengo, Verona, Italy; 23 Combined Department of Emergency, Urgency and Admission, Cattinara University Hospital, Trieste, Italy; 24 Department of Oncology and Onco-Hematology, University of Milan, Milan, Italy; 25 Department of Anaesthesia, Perioperative Medicine, Pain Therapy, RRS and Critical Care Area - DEA ASL2 Abruzzo, Chieti University Hospital, Chieti, Italy

**Keywords:** Anesthesia, Intraoperative care, Pneumonectomy, Postoperative care, Practice guideline, Thoracic surgery

## Abstract

**Introduction:**

Anesthetic care in patients undergoing thoracic surgery presents specific challenges that require a multidisciplinary approach to management. There remains a need for standardized, evidence-based, continuously updated guidelines for perioperative care in these patients.

**Methods:**

A multidisciplinary expert group, the Perioperative Anesthesia in Thoracic Surgery (PACTS) group, was established to develop recommendations for anesthesia practice in patients undergoing elective lung resection for lung cancer. The project addressed three key areas: preoperative patient assessment and preparation, intraoperative management (surgical and anesthesiologic care), and postoperative care and discharge. A series of clinical questions was developed, and literature searches were performed to inform discussions around these areas, leading to the development of 69 recommendations. The quality of evidence and strength of recommendations were graded using the United States Preventive Services Task Force criteria.

**Results:**

Recommendations for intraoperative care focus on airway management, and monitoring of vital signs, hemodynamics, blood gases, neuromuscular blockade, and depth of anesthesia. Recommendations for postoperative care focus on the provision of multimodal analgesia, intensive care unit (ICU) care, and specific measures such as chest drainage, mobilization, noninvasive ventilation, and atrial fibrillation prophylaxis.

**Conclusions:**

These recommendations should help clinicians to improve intraoperative and postoperative management, and thereby achieve better postoperative outcomes in thoracic surgery patients. Further refinement of the recommendations can be anticipated as the literature continues to evolve.

## Introduction

Thoracic surgery requires an evidence-based multidisciplinary approach that extends across the perioperative period, from preadmission evaluation to postoperative care and discharge. Although such perioperative care protocols, known as the enhanced recovery after surgery (ERAS^®^) “philosophy,” have been developed in many surgical settings, including lung surgery (Batchelor et al. 2019), and have been shown to be effective in reducing postoperative complications and length of hospital stay (LOS) (Nicholson et al. 2014), systematic reviews of studies in thoracic surgery (Cerfolio et al. 2001a; Das-Neves-Pereira et al. 2009; Muehling et al. 2008; Salati et al. 2012) have highlighted significant heterogeneity and methodological flaws in many trials (Fiore Jr et al. 2016; Li et al. 2017). To address this, an Italian expert group, the Perioperative Anesthesia Care in Thoracic Surgery (PACTS) group, was convened to develop evidence-based recommendations for the management of thoracic surgery patients.

## Methods

The PACTS group is a joint task force of the Italian Society of Anesthesia, Analgesia, Resuscitation, and Intensive Care (Società Italiana di Anestesia Analgesia Rianimazione e Terapia Intensiva, SIAARTI), The Italian Society of Thoracic Surgery (Società Italiana di Chirurgia Toracica, SICT), The Italian Society of Thoracic Endoscopy (Società Italiana di Endoscopia Toracica, SIET), The Italian Society of Surgery (Società Italiana di Chirurgia, SIC), The Italian Association of Hospital Pulmonologists (Associazione Italiana Pneuomologi Ospedalieri, AIPO), The Italian Society of Pneumology (Società Italiana di Pneumologia, SIP/IRS), and the VATS Group. The group comprises anesthetists, pulmonologists, thoracic surgeons, a clinical epidemiologist, and management staff.

The methods used to develop the PACTS recommendations have been described in full in an accompanying paper. In brief, the project focused on preoperative patient assessment and preparation, intraoperative management (surgical and anesthesiologic), and postoperative procedures and discharge in adult patients undergoing elective lung resection for lung cancer. A series of clinical questions were framed using the PICO (patients, intervention, comparison, outcome) approach, and a Delphi consensus method was used to reach agreement based on comprehensive literature searches. The quality of evidence and strength of recommendations were graded according to the United States Preventive Services Task Force (USPSTF) criteria (United States Preventive Services Task Force 2019); in addition, the panel classified as “Best Practice” the Recommendations considered to have a high level of certainty despite a lack of direct evidence. For USPSTF grade A, B, or C recommendations, consensus required > 70% A/B/C ratings with < 15% D/I ratings. For grade D or I recommendations, consensus required > 70% D/I ratings and < 15% A/B/C ratings. The USPSTF system was used in preference to the GRADE system, which has been used in the ERAS lung surgery guidelines (Batchelor et al. 2019), because the intention was to produce a position statement rather than full practice guidelines. The GRADE system involves full appraisal of a limited number of PICO questions, and is therefore time- and resource-consuming. It is not always feasible where a number of recommendations are required in fields where no large evidence base exists, or which cannot easily be addressed using a PICO framework.

Each author approved the final version prior to submission. This paper summarizes the final recommendations for intraoperative and postoperative care (Table 1), and the supporting evidence for each recommendation. The recommendations for preadmission and preoperative care are presented in the accompanying paper.

**Table 1:** List of recommendations for intraoperative and postoperative care

Recommendation	Level of evidence^a^	Strength of recommendation^a^
Intraoperative care		
The use of videolaryngoscopy for tracheal intubation with a double-lumen tube might improve visualization of the glottis and the success rate at the first attempt, reducing difficulty and positioning time. Videolaryngoscopy can be used in cases of unexpected difficult intubation.	Poor	C
We recommend the use of a double-lumen tube to manage one-lung ventilation. A single lumen tube with a bronchial blocker, rather than a double-lumen tube, is recommended for patients with difficult airways.	Good	A
We recommend the use of a flexible bronchoscope to control the position of the lung isolation device. Flexible bronchoscopy must always be available, even if not used routinely. Thoracic anesthesiologists must have adequate bronchoscopy skills to manage DLT and bronchial blockers for one-lung ventilation.	Good	A
We recommend monitoring arterial blood pressure with invasive (intra-arterial) techniques, rather than the non-invasive oscillometric cuff technique, in patients undergoing major thoracic surgery, or when sudden changes in hemodynamics, hemoglobin and blood gas concentrations (oxygen and carbon dioxide) are expected.	Good	A
We suggest considering the use of a central venous catheter on a case-by-case basis in patients undergoing thoracic surgery. Peripheral catheters are safe for short-term and low-dose treatment with inotropic vasoactive drugs.	Fair	C
In patients undergoing thoracic surgery who are considered at higher risk of postoperative complications, we suggest the use of hemodynamic monitoring with cardiac output estimation systems.	Poor	C
We do not recommend the use of dynamic preload indices during open-chest thoracic surgery, because these parameters might not be reliable.	Good	D
We suggest that patients undergoing thoracic surgery under general anesthesia are monitored with processed electroencephalography (pEEG) in order to titrate anesthetic administration.	Fair	B
We recommend that intraoperative temperature be monitored using an appropriate system in all patients undergoing thoracic surgery lasting more than 30 minutes. A core temperature of at least 36 °C should be maintained.	Good	A
We recommend monitoring neuromuscular blockade in all patients receiving neuromuscular blocking agents during general anesthesia for thoracic surgery.	Good	A
In low risk patients (simple procedures, younger patients and without cardiac or renal comorbidities), the use of a bladder catheter is not recommended.	Fair	D
We recommend using balanced crystalloid solutions, rather than normal saline (NaCl 0.9%), as standard fluid of choice.	Good	A
We do not recommend the use of hydroxyethyl starch as routine fluid therapy in patients undergoing thoracic surgery.	Good	D
We recommend a near-zero, rather than restricted or permissive, fluid balance to patients undergoing thoracic surgery. In high-risk patients a goal-directed approach to fluid therapy should be applied.	Fair	A
We suggest using serum hemoglobin concentration in the evaluation of volume status in non-bleeding patients undergoing thoracic surgery.	Poor	C
We recommend a protective ventilation approach during one-lung ventilation, based on the combination of low tidal volumes (≤ 6 mL/kg ideal body weight) with alveolar recruitment maneuvers, adequately titrated positive end-expiratory pressure (PEEP) and the lowest fraction of inspired oxygen (FiO2) to maintain satisfactory arterial oxygen saturation.	Fair	A
Volatile anesthesia cannot be recommended over intravenous propofol administration in order to reduce postoperative complications, although there is evidence of a lower degree of both systemic and local inflammation when volatile anesthetics are used.	Good	I
We recommend the use of a steroid neuromuscular blocking agent because of the availability of sugammadex, a reversal agent that, unlike acetylcholinesterase inhibitors, can be used even in cases of deep residual block, and reduces both extubation time and adverse events (bradycardia, postoperative nausea and vomiting and postoperative residual paralysis).	Fair	A
We recommend evaluation of the risk of postoperative nausea and vomiting, and the use of appropriate prophylaxis according to the level of risk, in all patients undergoing lung surgery.	Good	A
We recommend avoiding the routine placement of a nasogastric tube, and early removal in patients in whom a nasogastric tube is used.	Fair	A
We recommend the early removal of urinary catheters to promote mobilization in patients undergoing lung surgery, including those receiving thoracic epidural catheters.	Fair	A
Postoperative care		
We recommend the use of pre-emptive locoregional analgesia as part of a multimodal analgesic approach for thoracic surgery. Systemic opioids, nonsteroidal anti-inflammatory drugs, and paracetamol have shown no evidence of benefit when used as pre-emptive analgesics.	Fair	A
Currently, there are no elements to suggest the routine perioperative use of gabapentinoids in patients undergoing thoracic surgery, but their use can be effective in a comprehensive multimodal analgesia protocol.	Poor	I
We suggest intraoperative intravenous administration of ketamine to reduce postoperative pain after thoracic surgery. There is no evidence about the best dose and timing of administration of ketamine.	Fair	B
We suggest intraoperative intravenous administration of magnesium sulfate to reduce postoperative pain after thoracic surgery.	Fair	B
There is no evidence to suggest the routine use of α_2_-agonists as part of a multimodal analgesia regimen to reduce postoperative pain after thoracic surgery. There is no consensus on the best timing and schedule for administration of these drugs.	Fair	I
We suggest considering the use of intravenous steroids as part of a multimodal approach to reduce peripheral sensibilization of inflammatory-induced pain in patients undergoing thoracic surgery. Adverse effects of single doses of steroids are of trivial clinical impact.	Fair	C
We recommend the use of intravenous nonsteroidal anti-inflammatory drugs (NSAIDs) to reduce peripheral sensitization to inflammation-induced pain in patients undergoing thoracic surgery. Combined use of NSAIDs and paracetamol may give a further analgesic advantage.	Good	A
We recommend the use of locoregional anesthesia for intraoperative and postoperative pain management.	Poor	A
We recommend the use of thoracic epidural analgesia in high-risk patients or in major surgical procedures where the parietal pleura (eg chest wall resection) is violated (i.e. thoracotomy, thoracosternotomy, chest wall resection).	Fair	A
We recommend thoracic paravertebral block for VATS, as part of a multimodal approach.	Good	A
We recommend paravertebral block in preference to thoracic epidural analgesia in patients with known or suspected coagulopathy.	Fair	A
We suggest that intercostal nerve blockade should be considered only as a second choice for analgesia after thoracic surgical procedures.	Good	C
We suggest erector spinae plane block as part of a multimodal analgesia for thoracic surgery, especially for VATS.	Poor	B
We suggest the use of fascial pain blocks as part of multimodal analgesia for thoracic surgery, particularly for VATS.	Fair	B
We suggest considering the use of adjuvants (i.e. opioids, clonidine, dexmedetomidine^b^, dexamethasone, magnesium) when loco-regional anesthesia is performed, because the use of adjuvants can potentiate and prolong the effect of local anesthetics.	Poor	C
We suggest considering the use of a single large-bore chest tube instead of a double tube after thoracic surgery. Insertion of more than one chest tube may be considered in selected cases (e.g., bi-lobectomy or bleeding patients).	Poor	C
We suggest considering the use of digital chest drainage systems to promote early mobilization of the patient.	Fair	B
The routine use of drainage with suction is not recommended in the absence of complications, provided there is full re-expansion of the residual parenchyma after lung resection.	Good	D
We suggest removing chest tubes in lung resection patients when liquid output is ≤ 5 cm^3^/kg/24 h of serous fluid.	Poor	B
We do not recommend systematic ICU admission after thoracic surgery.	Poor	D
We recommend that, in adult patients undergoing thoracic surgery, oral intake, including clear liquids, can be initiated 4-6 hours after surgery, in the absence of nausea and vomiting. Oral intake should, however, be adapted to individual tolerance.	Fair	A
We recommend early mobilization of patients within the first 24 h after both minor and major thoracic surgery.	Fair	A
We recommend a physiotherapy program after thoracic surgery.	Fair	A
We suggest considering daily chest radiographs only in selected cases under specific clinical indications.	Good	C
We do not recommend the routine use of either continuous positive airway pressure (CPAP) or non invasive ventilation (NIV) to prevent postoperative pulmonary complications, prolonged length of stay, and mortality (both in ICU and in hospital) in patients undergoing major thoracic surgery. CPAP or NIV could be considered case by case in selected high risk patients.	Poor	D
We suggest the use of NIV or CPAP to treat acute respiratory failure complicating thoracic surgery.	Poor	B
We suggest considering the use of high-flow nasal cannula oxygen therapy (HFNC) as an alternative or integrative support to CPAP or NIV to prevent or treat acute respiratory failure complicating thoracic surgery.	Poor	C
For prophylaxis and management of atrial fibrillation after thoracic surgery, we recommend reference to the Society of Thoracic Surgery (STS) 2011 Guidelines.	Good	A

^a^Level of evidence and strength of recommendation were rated according to the United States Preventive Services Task Force (USPSTF) criteria (United States Preventive Services Task Force 2019)

^b^Dexmedetomidine is currently approved in Italy only for sedation, and thus cannot be recommended for analgesic use in Italian settings

## Intraoperative care

### Airway management

Recommendation 1: The use of videolaryngoscopy for tracheal intubation with a double-lumen tube might improve visualization of the glottis and the success rate at the first attempt, reducing difficulty and positioning time. Videolaryngoscopy can be used in cases of unexpected difficult intubation.

Level of evidence: Poor

Strength of recommendation: C

Several studies have compared videolaryngoscopy with the Macintosh blade laryngoscope for tracheal intubation, in order to determine whether videolaryngoscopy improves the speed and success of double-lumen tube (DLT) positioning and reduces malpositioning rates (El-Tahan et al. 2018; Hamp et al. 2015; Lin et al. 2012; Purugganan et al. 2012; Russell et al. 2013; Wasem et al. 2013). These studies have yielded conflicting results: while some authors have reported that videolaryngoscopy is superior to the Macintosh laryngoscope blade in terms of ease of use and higher rates of correct positioning of the DLT (Lin et al. 2012; Purugganan et al. 2012), others have found no significant differences between the two techniques in terms of time to intubation and hemodynamic stress response (Hamp et al. 2015; Russell et al. 2013; Wasem et al. 2013). There are limited data to suggest that videolaryngoscopy may improve visualization of the glottis, resulting in higher success rates at the first attempt, and reduced difficulty and positioning time (Lin et al. 2012; Purugganan et al. 2012). However, the success rate is highly dependent on the operator’s experience (El-Tahan et al. 2018).

Recommendation 2: We recommend the use of a double-lumen tube to manage one-lung ventilation. A single lumen tube with a bronchial blocker, rather than a double-lumen tube, is recommended for patients with difficult airways.

Level of evidence: Good

Strength of recommendation: A

Lung isolation techniques are designed to facilitate surgical exposure of the lung and achieve one-lung ventilation in patients undergoing thoracic surgery (Campos and Kernstine 2003; Narayanaswamy et al. 2009). These techniques use either a DLT with both an endotracheal and an endobronchial lumen, or a bronchial blocker inside a single-lumen endotracheal tube, which allows collapse of the lung distal to the site of occlusion. DLTs offer a number of advantages over bronchial blockers, including faster and easier positioning (Campos and Kernstine 2003; Narayanaswamy et al. 2009; Clayton-Smith et al. 2015; Dumans-Nizard et al. 2009; Ruetzler et al. 2011), and a lower likelihood of displacement requiring repositioning under bronchoscopy (Campos and Kernstine 2003; Narayanaswamy et al. 2009). In addition, pulmonary collapse can be achieved more quickly with DLTs, because bronchial blockers do not allow adequate suction to cause lung collapse (Campos 2002; Yoo et al. 2014). DLTs also ensure pulmonary isolation, protecting the contralateral lung from blood or infections (Santana-Cabrera et al. 2010), although the incidence of trauma during intubation is comparable with the two types of device (Clayton-Smith et al. 2015; Knoll et al. 2006). For these reasons, SIAARTI guidelines recommend DLTs for routine clinical use (Merli et al. 2009). The decision to use a bronchial blocker, rather than a DLT, in an individual patient should be based on the specific clinical circumstances (Merli et al. 2009; Campos 2007).

Recommendation 3: We recommend the use of a flexible bronchoscope to control the position of the lung isolation device. Flexible bronchoscopy must always be available, even if not used routinely. Thoracic anesthesiologists must have adequate bronchoscopy skills to manage DLT and bronchial blockers for one-lung ventilation.

Level of evidence: Good

Strength of recommendation: A

The use of a flexible bronchoscope to confirm the correct placement of DLTs for lung resection is recommended. Studies have shown that flexible bronchoscopy after auscultatory or tactile confirmation of the location of the DLT can identify malpositioning in more than one-third of patients (Klein et al. 1998; de Bellis et al. 2011), and hence some authors have recommended that the position of the DLT should routinely be confirmed by fiberoptic bronchoscopy (Klein et al. 1998; Cohen 2004). However, this requires technical expertise in flexible bronchoscopy, and a detailed knowledge of tracheobronchial anatomy (Cohen 2004; Campos 2009; Solidoro et al. 2019). It remains unclear whether routine bronchoscopic confirmation of the position of the DLT is necessary.

Malpositioning of the DLT is a major cause of intraoperative hypoxemia: in one case series, 21 of 56 patients in whom the DLT was positioned too deeply in the left bronchus developed hypoxemia during one-lung ventilation of the left lung (Brodsky and Lemmens 2003). For this reason, the position of the DLT must be rechecked by flexible bronchoscopy after the onset of intraoperative hypoxemia, with the patient in the lateral decubitus position (Brodsky and Lemmens 2003; Inoue et al. 2004).

Obstruction of the left or right upper lobe bronchus is the most common significant malposition with DLTs (Slinger 1989), but there is no consensus as to the optimal position of the DLT. Many malpositions may be attributable to an inappropriate choice of DLT or suboptimal positioning technique (Slinger 1989; Fortier et al. 2001; Seymour and Lynch 2002). To date, no data have demonstrated the clinical relevance of malpositioning to patient outcomes, except in cases of dangerous or critical malposition, and there is no evidence that routine confirmation of DLT positioning by flexible bronchoscopy reduces morbidity after thoracic surgery.

When a left DLT is inserted, the use of tubes with integrated high-resolution cameras can facilitate correct positioning and easier one-lung ventilation (Massot 2015; Schuepbach et al. 2015). In one study, the mean time to successful intubation was significantly shorter with the VivaSight-DL (ETView Medical Ltd, Misgav, Israel) than with conventional DLTs (63 s versus 97 s, respectively, *P* = 0.03), and all VivaSight-DL tubes were correctly positioned (Schuepbach et al. 2015). Furthermore, compared with blind placement, the use of tubes with integrated high-resolution cameras can shorten the intubation time and permits continued monitoring of the carina, thereby allowing prompt management of intraoperative tube displacement (Massot 2015; Schuepbach et al. 2015; Belze et al. 2017; Chen et al. 2017; Heir et al. 2014).

### Patient monitoring

Recommendation 4: We recommend monitoring arterial blood pressure with invasive (intra-arterial) techniques, rather than the non-invasive oscillometric cuff technique, in patients undergoing major thoracic surgery, or when sudden changes in hemodynamics, hemoglobin and blood gas concentrations (oxygen and carbon dioxide) are expected.

Level of evidence: Good

Strength of recommendation: A

Limited data suggest good concordance between invasive and non-invasive arterial pressure measurements in patients undergoing major thoracic surgery (Bardoczky et al. 1992; D'Antini et al. 2016; Martina et al. 2012), but further studies are needed in this area. Due to the possibility of rapid changes in stroke volume and arterial blood pressure, and the potential usefulness of arterial blood sampling for gas, hemoglobin, and electrolyte analysis, invasive (intra-arterial) monitoring of arterial blood pressure is recommended in patients undergoing major thoracic surgery. In general, the risk of significant blood loss is very low in patients with no history of radiotherapy or chemotherapy who are undergoing primary lung surgery. For patients undergoing minor resections, the use of invasive blood pressure monitoring should be considered on a case-by-case basis according to the patient’s comorbidity and surgical complexity.

Although specific studies on thoracic surgery patients are lacking, studies in mixed surgical populations have demonstrated that even short periods of hypotension significantly increase postoperative complications such as acute kidney injury (AKI), myocardial injury after non-cardiac surgery (MINS), and death (van Waes et al. 2016; Walsh et al. 2013; Sessler et al. 2019). In a review of data from 33,330 patients undergoing non-cardiac surgery, the relative risks of both AKI and MINS increased progressively with increasing duration of hypotension (mean arterial pressure < 55 mmHg), compared with patients with mean arterial pressure above this threshold, even when the duration of hypotension was only 1–5 min (Walsh et al. 2013). A mean arterial pressure threshold of 65 mmHg, or 60–70 mmHg with a systolic arterial pressure of 100 mmHg, has been identified as critical to reduce the occurrence of AKI, MINS and mortality (Sessler et al. 2019; Salmasi et al. 2017).

Recommendation 5: We suggest considering the use of a central venous catheter on a case-by-case basis in patients undergoing thoracic surgery. Peripheral catheters are safe for short-term and low-dose treatment with inotropic vasoactive drugs.

Level of evidence: Fair

Strength of recommendation: C

There is no evidence that central venous catheters are essential for the intraoperative and postoperative management of thoracic surgery patients. Measurement of central venous pressure to predict the response to volume expansion may be inconclusive in a significant proportion of patients (Cannesson et al. 2011). Furthermore, several studies have shown that low doses of vasoactive medications can be safely administered via peripheral intravenous catheters, with extravasation rates of approximately 2–4% (Cardenas-Garcia et al. 2015; Lewis et al. 2019; Medlej et al. 2018). For these reasons, the routine use of central venous catheters is not recommended in patients undergoing thoracic surgery: the need for central venous catheterization should be evaluated on a case-by-case basis.

Recommendation 6: In patients undergoing thoracic surgery who are considered at higher risk of postoperative complications, we suggest the use of hemodynamic monitoring with cardiac output estimation systems.

Level of evidence: Poor

Strength of recommendation: C

There is evidence that hemodynamic monitoring using cardiac output estimation systems to inform goal-directed fluid management is beneficial in thoracic surgery patients at higher risk of postoperative complications (Cecconi et al. 2013; Kaufmann et al. 2017; Michard et al. 2017; Searl and Perrino 2012; Zhang et al. 2013). Furthermore, such monitoring can be useful to avoid hypoxemia during one-lung ventilation, because extreme increases or decreases in cardiac output can impair the hypoxic pulmonary vasoconstriction (Lumb and Slinger 2015). The use of this approach should be based on the estimated risk of complications in the individual patient.

Recommendation 7: We do not recommend the use of dynamic preload indices during open-chest thoracic surgery, because these parameters might not be reliable.

Level of evidence: Good

Strength of recommendation: D

A recent meta-analysis of seven trials has found that pulse pressure and stroke volume are inaccurate predictors of fluid responsiveness in patients undergoing open thoracotomy (Piccioni et al. 2017), and a subsequent study has shown that this is also true in patients undergoing VATS procedures (Jeong et al. 2017).

Recommendation 8: We suggest that patients undergoing thoracic surgery under general anesthesia are monitored with processed electroencephalography (pEEG) in order to titrate anesthetic administration*.*

Level of evidence: Fair

Strength of recommendation: B

Processed electroencephalography (pEEG) based on bispectral index (BIS) reduces recovery times (Punjasawadwong et al. 2014; Chiang et al. 2018). However, the impact of pEEG on the risk of intraoperative awareness is unclear (Punjasawadwong et al. 2014). Postoperative delirium occurs in approximately 14% of patients (Berian et al. 2018), and it is believed that monitoring the depth of anesthesia by pEEG is associated with reductions in the incidence of postoperative delirium and cognitive dysfunction (POCD) (Aldecoa et al. 2017; Fritz et al. 2016). A recent meta-analysis of six randomized controlled trials showed moderate-quality evidence that pEEG-guided anesthesia could reduce the risk of postoperative delirium and POCD (Punjasawadwong et al. 2018). Conversely, the ENGAGES study, a RCT of 1232 patients undergoing major surgery under volatile general anesthesia, did not find any decrease in the incidence of postoperative delirium among patients managed with pEEG, compared with usual care (Wildes et al. 2019). pEEG has been included in guidelines for the prevention of postoperative delirium from a number of organizations (Aldecoa et al. 2017; *J Am Coll Surg* 2015; Gelb et al. 2018). Advanced pEEG technology is considered useful to improve anesthesia monitoring, individual titration of anesthetics and optimized patient care (Eagleman and Drover 2018; Fahy and Chau 2018; Montupil et al. 2019).

Recommendation 9: We recommend that intraoperative temperature be monitored using an appropriate system in all patients undergoing thoracic surgery lasting more than 30 min. A core temperature of at least 36 °C should be maintained.

Level of evidence: Good

Strength of recommendation: A

Hypothermia occurs in approximately 35–50% of thoracic surgery patients because the pleural surface on one side of the thorax is exposed to dry air during surgery, leading to evaporative heat loss (Batchelor et al. 2019). Avoidance of hyperthermia is essential to prevent deleterious effects on homeostasis and reduce the risk of a systemic inflammatory response. Hence, the ERAS guidelines for thoracic surgery recommend that body temperature should be continuously monitored to guide therapy, and that active warming should be continued postoperatively until the patient’s temperature is greater than 36 °C (Batchelor et al. 2019).

SIAARTI guidelines recommend that intraoperative temperature should be monitored in all patients undergoing thoracic surgery lasting more than 30 min, and that the aim should be to maintain a core temperature of at least 36 °C (Di Marco and Cannetti 2019). Suitable monitoring systems include heated servo-controlled sensors, intra-vascular catheters with thermistor tips, or rectal or bladder probes, but esophageal probes may be less accurate (Di Marco and Cannetti 2019). A number of studies in various surgical settings have found that zero-heat-flux systems can be used for non-invasive temperature measurement, and show good agreement with conventional core temperature measurements (Eshraghi et al. 2014; Iden et al. 2015; Makinen et al. 2016).

Recommendation 10: We recommend monitoring neuromuscular blockade in all patients receiving neuromuscular blocking agents during general anesthesia for thoracic surgery.

Level of evidence: Good

Strength of recommendation: A

Neuromuscular blockade should be monitored in all patients receiving neuromuscular blocking agents (NMBAs) during general anesthesia for thoracic surgery (Ortega et al. 2018). Quantitative (objective) neuromuscular monitoring is more reliable than subjective and clinical tests to assess the neuromuscular block level and, more importantly, recovery before extubation (Naguib et al. 2018). Neuromuscular monitoring is essential for correct administration of both NMBAs and reversal agents.

Recommendation 11: In low-risk patients (simple procedures, younger patients and without cardiac or renal comorbidities), the use of a bladder catheter is not recommended.

Level of evidence: Fair

Strength of recommendation: D

There is no evidence that urine output should be monitored in all patients undergoing thoracic surgery.

### Anesthesia management

Recommendation 12: We recommend using balanced crystalloid solutions, rather than normal saline (NaCl 0.9%), as standard fluid of choice.

Level of evidence: Good

Strength of recommendation: A

Balanced crystalloid solutions differ from normal saline (NaCl 0.9%) in that they contain anions other than chloride, such as lactate, acetate, malate, and gluconate, which act as physiological buffers (Reddy et al. 2016; Vincent and De Backer 2016). Although specific studies in thoracic surgery patients are lacking, the available evidence suggests that normal saline is associated with risks of hyperchloremia, hyperchloremic acidosis and AKI (Reddy et al. 2016; Zampieri et al. 2016). For example, in a study in noncritically ill patients, the 30-day incidence of major renal adverse events in patients receiving balanced crystalloids or saline was 4.7% and 5.6%, respectively (odds ratio [OR] 0.82, 95% confidence interval [CI] 0.70–0.95, *P* = 0.01), although there was no difference in the number of hospital-free days between the two treatments (Self et al. 2018). In general, most authors recommend that balanced crystalloids should be used in preference to normal saline (Reddy et al. 2016; Vincent and De Backer 2016). Administration of normal saline is indicated only in specific circumstances, such as metabolic alkalosis, hyponatremia, or severe brain injury requiring normotonic fluid administration (Vincent and De Backer 2016).

Recommendation 13: We do not recommend the use of hydroxyethyl starch as routine fluid therapy in patients undergoing thoracic surgery.

Level of evidence: Good

Strength of recommendation: D

Patients undergoing lung resection surgery are at risk of postoperative respiratory failure, which could be related to the volume of fluid administered during surgery. Hydroxyethyl starches could be administered in order to reduce the total amount of fluid given during surgery, but are associated with an increased risk of renal impairment (Ahn et al. 2016). Hence, the use of hydroxyethyl starch as routine fluid therapy should be avoided in patients undergoing thoracic surgery, although it could be considered in patients with severe hemorrhage who are not responding to crystalloid infusion (De Hert and De Baerdemaeker 2014).

Recommendation 14: We recommend a near-zero, rather than restricted or permissive, fluid balance to patients undergoing thoracic surgery. In high-risk patients, a goal-directed approach to fluid therapy should be applied.

Level of evidence: Fair

Strength of recommendation: A

There is evidence that a near-zero, rather than restricted or permissive, fluid balance is beneficial for patients undergoing thoracic surgery (Searl and Perrino 2012), and hence this approach is recommended in normovolemic patients (Chappell et al. 2008; Licker et al. 2016). In high-risk patients, a goal-directed approach to fluid therapy is recommended because this has been shown to significantly reduce mortality and morbidity, compared with standard hemodynamic fluid management (Cecconi et al. 2013; Kaufmann et al. 2017; Michard et al. 2017; Zhang et al. 2013). For example, a recent meta-analysis of 19 trials involving over 2000 patients found that goal-directed therapy was associated with a significant decrease in postoperative morbidity, compared with controls (OR 0.46, 95% CI 0.30–0.70, *P* < 0.001) (Michard et al. 2017). Similarly, a meta-analysis of 32 trials involving approximately 2800 patients found a significant reduction in postoperative mortality with goal-directed therapy, compared with controls, in patients at highest risk of postoperative complications (OR 0.20, 95% CI 0.09–0.41, *P* < 0.0001); there was also a significant reduction in complication rates (OR 0.45, 95% CI 0.34–0.60, *P* < 0.00001), which was particularly marked in the highest risk subgroup (OR 0.27, 95% CI 0.15–0.51, *P* < 0.0001) (Cecconi et al. 2013).

Recommendation 15: We suggest using serum hemoglobin concentration in the evaluation of volume status in non-bleeding patients undergoing thoracic surgery.

Level of evidence: Poor

Strength of recommendation: C

Because hemoglobin concentrations reflect plasma volume changes in patients without significant bleeding, monitoring of hemoglobin levels may play a role in the evaluation of volume status in patients undergoing thoracic surgery (Perel 2017; Otto et al. 2017).

Recommendation 16: We recommend a protective ventilation approach during one-lung ventilation, based on the combination of low tidal volumes (≤ 6 mL/kg ideal body weight) with alveolar recruitment maneuvers, adequately titrated positive end-expiratory pressure (PEEP) and the lowest fraction of inspired oxygen (FiO_2_) to maintain satisfactory arterial oxygen saturation.

Level of evidence: Fair

Strength of recommendation: A

Although there is an emerging consensus in favor of protective ventilation during one-lung ventilation (Lohser and Slinger 2015), relatively few well-designed randomized trials have compared protective and conventional one-lung ventilation (Lohser and Slinger 2015; Ahn et al. 2012; Kim et al. 2019; Yang et al. 2011; Zhu et al. 2017): most published studies have involved small patient populations, or had other methodological limitations. In one of the largest randomized trials, 100 patients undergoing elective lobectomy were randomized to receive either protective ventilation with an inspired oxygen fraction (FiO_2_) of 0.5, a tidal volume of 6 mL/kg, a positive end-expiratory pressure (PEEP) of 5 cm H_2_O, and pressure-controlled ventilation, or conventional ventilation with higher FiO_2_ and tidal volume, zero end-expiratory pressure, and volume-controlled ventilation (Yang et al. 2011). The incidence of pulmonary dysfunction (defined as PaO_2_/FiO_2_ < 300 mmHg, lung infiltration or atelectasis) was significantly lower in patients receiving protective ventilation than in those receiving conventional ventilation (4% versus 22% respectively, *P* < 0.05). A further randomized trial, involving 65 patients undergoing VATS lobectomy, found no significant difference in postoperative complication rates between patients receiving either volume-controlled or pressure-controlled protective ventilation (Zhu et al. 2017). By contrast, a randomized study in 50 patients found that protective ventilation did not offer any significant advantage, compared with conventional ventilation, in terms of postoperative pulmonary dysfunction (PaO_2_/FiO_2_ < 300 mmHg or radiographic abnormalities) in patients undergoing VATS (Ahn et al. 2012).

Further evidence supporting the use of protective ventilation in thoracic surgery patients comes from observational studies (Blank et al. 2016; Okahara et al. 2018). In a review of data from 1019 thoracic surgery patients (Blank et al. 2016), there was an inverse relationship between tidal volume and the incidence of respiratory complications (OR 0.84, 95% CI 0.73–0.96); however, a low (physiologically appropriate) tidal volume had no protective effect in the absence of an adequate PEEP. A further study found that FiO_2_ during one-lung ventilation was an independent predictor of the risk of postoperative pulmonary complications: the risk of such complications increased by 30% for each 0.1 increase in FiO_2_ (Okahara et al. 2018).

Two small studies have examined the effect of protective ventilation on inflammatory responses following one-lung ventilation. A small randomized study in VATS patients found that the combination of protective ventilation with a recruitment maneuver was associated with attenuated inflammatory responses, compared with either conventional ventilation or protective ventilation alone (Kim et al. 2019). By contrast, a non-randomized study in 28 patients found no significant difference in local inflammatory cytokine responses between lung resection patients receiving protective or conventional ventilation (Fiorelli et al. 2018).

Recommendation 17: Volatile anesthesia cannot be recommended over intravenous propofol administration in order to reduce postoperative complications, although there is evidence of a lower degree of both systemic and local inflammation when volatile anesthetics are used.

Level of evidence: Good

Strength of recommendation: I

The clinical impact of the choice of anesthetic in thoracic surgery patients is unclear because published studies differ markedly in their design, and have yielded conflicting findings. It has been suggested that only patients with severe surgical injuries (i.e., those undergoing pneumonectomy) may benefit clinically from the anti-inflammatory effects of volatile anesthetics (Beck-Schimmer et al. 2016), but further studies are needed to clarify this.

Several studies have compared the use of volatile halogenated anesthesia and intravenous propofol administration, most of which have found that volatile anesthetics are associated with a lower degree of alveolar—and possibly systemic—inflammatory responses (De Conno et al. 2009; de la Gala et al. 2017; Potocnik et al. 2014; Schilling et al. 2011; Sun et al. 2015). In a meta-analysis of eight randomized controlled trials in patients undergoing one-lung ventilation, volatile anesthetics were associated with significant decreases, compared with intravenous anesthetics, in alveolar concentrations of inflammatory mediators (Sun et al. 2015). Other studies have shown that, compared with propofol, the volatile halogenated anesthetics desflurane and sevoflurane reduce the expression of inflammatory mediators in bronchoalveolar lavage fluid, and the inflammatory response of alveolar epithelial cells to one-lung ventilation; these effects may be attributable to protective effects on the endothelial glycocalyx (De Conno et al. 2009; de la Gala et al. 2017; Schilling et al. 2011; Duthie 2013; Schilling et al. 2007).

In contrast to the consistent evidence for anti-inflammatory effects of volatile anesthetics, studies of the effects of volatile or intravenous anesthetics on postoperative complications have yielded conflicting results, possibly due to differences in study designs and the definition of postoperative complications. Several studies have shown lower rates of postoperative pulmonary complications with volatile anesthetics, compared with propofol, in patients receiving one-lung ventilation (De Conno et al. 2009; de la Gala et al. 2017; Potocnik et al. 2014). In the meta-analysis cited above (Sun et al. 2015), the relative risk of pulmonary complications in patients receiving inhalation anesthetics, compared with those receiving intravenous anesthetics, was 0.42 (95% CI 0.23–0.77, *P* = 0.005), and the mean duration of hospitalization was approximately 4 days shorter. However, a recent large, multicenter, randomized trial involving 460 thoracic surgery patients found no significant difference in complication rates between patients receiving desflurane or propofol (Beck-Schimmer et al. 2016). The proportion of patients with major complications was 13.0% and 16.5%, respectively, during hospitalization (hazard ratio [HR] 0.75, 95% CI 0.46–1.22; *P* = 0.24) and 39.6% and 40.4%, respectively, at 6 months (HR 0.95, 95% CI 0.71–1.28, *P* = 0.71). Subgroup analyses suggested that only patients with severe surgical injuries benefit from the anti-inflammatory effects of volatile anesthetics (Beck-Schimmer et al. 2016).

Recommendation 18: We recommend the use of a steroid neuromuscular blocking agent because of the availability of sugammadex, a reversal agent that, unlike acetylcholinesterase inhibitors, can be used even in cases of deep residual block, and reduces both extubation time and adverse events (bradycardia, postoperative nausea and vomiting, and postoperative residual paralysis).

Level of evidence: Fair

Strength of recommendation: A

Deep neuromuscular blockade, with appropriate reversal prior to extubation, is recommended for patients undergoing thoracic surgery (Umari et al. 2018; Granell et al. 2018; Végh et al. 2014). Complete reversal of neuromuscular blockade after surgery is important because it facilitates ventilator movements and expectoration, thereby decreasing the risk of postoperative respiratory complications (Végh et al. 2014). The use of a steroid NMBA, such as rocuronium, with complete reversal, reduces the extubation time, compared with non-steroidal NMBAs (Carron et al. 2017; Hristovska et al. 2017).

The use of a selective relaxant-binding agent such as sugammadex is more efficient and safer than neostigmine for reversing moderate or deep induced paralysis (Flockton et al. 2008). In a prospective observational study involving 3000 patients, the use of neostigmine for reversal of neuromuscular blockade did not improve oxygenation at the time of admission to the post-anesthesia care unit, and was associated with a higher rate of atelectasis, compared with patients who did not receive neostigmine (8.8% versus 4.5%, OR 1.67, 95% CI 1.07–2.59) (Sasaki et al. 2014). In addition, high-dose neostigmine (> 60 μg/kg) was associated with longer stays in the post-anesthesia unit (mean 176 versus 157 min) and longer postoperative hospitalization (mean 2.9 versus 2.8 days). By contrast, a 2017 Cochrane review found that patients receiving sugammadex for reversal of neuromuscular blockade had 40% fewer adverse events (risk ratio [RR] 0.60, 95% CI 0.49–0.74), including less postoperative nausea and vomiting (PONV), bradycardia, or postoperative residual paralysis, than those receiving neostigmine (Hristovska et al. 2017). Furthermore, sugammadex produced faster reversal of neuromuscular blockade than neostigmine, irrespective of the depth of blockade (Hristovska et al. 2017).

Recommendation 19: We recommend evaluation of the risk of postoperative nausea and vomiting, and the use of appropriate prophylaxis according to the level of risk, in all patients undergoing lung surgery.

Level of evidence: Good

Strength of recommendation: A

There is a lack of specific data on PONV after thoracic surgery. Recently, a randomized controlled trial in patients undergoing VATS procedures showed a lower incidence of nausea on the day of surgery in patients receiving preoperative treatment with methylprednisolone, compared with placebo-treated patients, although there was no difference between the groups on postoperative days 1 and 2 (Bjerregaard et al. 2018). The 2014 Society for Ambulatory Anesthesia Guidelines for the Management of Postoperative Nausea and Vomiting recommend preoperative evaluation of PONV risk using validated scores, such as the simplified Apfel score, and the use of appropriate prophylaxis (Gan et al. 2014). Strategies to reduce the risk of PONV suggested in these guidelines include the use of propofol rather than volatile anesthetics, and minimization of intra- and postoperative opioids. Prophylaxis against PONV is also recommended in ERAS guidelines (Batchelor et al. 2019; Ljungqvist and Hubner 2018).

Recommendation 20: We recommend avoiding the routine placement of a nasogastric tube, and early removal in patients in whom a nasogastric tube is used.

Level of evidence: Fair

Strength of recommendation: A

Nasogastric tubes can be used to identify the esophagus, and to reduce gastric distension and risk of aspiration. There are no specific data in the literature on the use of nasogastric tubes in patients undergoing lung surgery, but several studies have identified perioperative nasogastric tube use as a risk factor for postoperative pulmonary complications after abdominal surgery (Miskovic and Lumb 2017). Guidelines published by the ERAS Society recommend avoiding routine nasogastric tube placement in patients undergoing liver and gastric surgery (Melloul et al. 2016; Mortensen et al. 2014), and the removal of nasogastric tubes before anesthesia reversal following elective colonic surgery (Gustafsson et al. 2013).

Recommendation 21: We recommend the early removal of urinary catheters to promote mobilization in patients undergoing lung surgery, including those receiving thoracic epidural catheters.

Level of evidence: Fair

Strength of recommendation: A

Monitoring of urine output to evaluate perioperative AKI is included in all classification systems for renal dysfunction (Goren and Matot 2015), but a large prospective observational study found no association between intraoperative oliguria (urine output < 0.5 mL/kg/h) and postoperative AKI in patients undergoing major noncardiac surgery (Kheterpal et al. 2007). Higher rates of urinary retention after early urinary catheter removal (within 24–48 h after surgery), compared with later removal, have been reported in patients who received epidural analgesia for pain management after thoracotomy (Allen et al. 2016; Hu et al. 2014), but other studies have found no association between early removal and increased complication rates (Chia et al. 2009; Ladak et al. 2009; Young et al. 2018). A systematic review recommended early removal of the urinary catheter, on the first postoperative day, in order to promote mobilization and reduce pain and discomfort (Zaouter and Ouattara 2015). Early removal of urinary catheters is one of the overall ERAS items intended to promote mobilization and ambulation (Ljungqvist and Hubner 2018). In addition, the ERAS guidelines for lung surgery strongly recommend not to routinely use urinary catheterization solely to monitor urine output in patients with normal kidney function, but to use a urinary catheter in patients receiving epidural analgesia (Batchelor et al. 2019).

## Postoperative care

### Pre-emptive analgesia

Recommendation 22: We recommend the use of pre-emptive locoregional analgesia as part of a multimodal analgesic approach for thoracic surgery. Systemic opioids, nonsteroidal anti-inflammatory drugs, and paracetamol have shown no evidence of benefit when used as pre-emptive analgesics.

Level of evidence: Fair

Strength of recommendation: A

Multiple studies in various surgical settings have shown that the use of pre-emptive locoregional analgesia attenuates postoperative pain scores, decreases supplemental analgesic requirements, and prolongs the average time to first use of rescue analgesia (Nosotti et al. 2015; Ong et al. 2005; Yang et al. 2015). As a result, pre-emptive locoregional analgesia is recommended as part of a multimodal analgesic strategy for thoracic surgery patients. There is currently no evidence to support the use of one form of analgesia (opioids, nonsteroidal anti-inflammatory drugs [NSAIDs], paracetamol, etc) over another.

Recommendation 23: Currently, there are no elements to suggest the routine perioperative use of gabapentinoids in patients undergoing thoracic surgery, but their use can be effective in a comprehensive multimodal analgesia protocol.

Level of evidence: Poor

Strength of recommendation: I

Studies evaluating gabapentin in thoracic surgery patients are limited, and have yielded conflicting results. A randomized, active placebo (lorazepam)-controlled, trial in a mixed surgical cohort found that perioperative gabapentin administration until the third postoperative day had no effect on the time to cessation of acute postoperative pain (HR 1.04, 95% CI 0.82–1.33, *P* = 0.73), but had a moderate effect on the time to opioid cessation (HR 1.24, 95% CI 1.00–1.54, *P* = 0.05) (Hah et al. 2018). Two further studies found no benefit of gabapentin treatment, in terms of postoperative pain relief, opioid consumption, and the incidence of chronic pain 3 months after thoracotomy (Grosen et al. 2014; Kinney et al. 2012); similarly, a small randomized trial found that gabapentin had no significant effect, compared with placebo, on the incidence or severity of post-thoracotomy shoulder pain (Huot et al. 2008). On the basis of such findings, a 2013 review concluded that there is no evidence to support the role of a single preoperative oral dose of gabapentin in reducing pain scores or opioid consumption following thoracic surgery (Zakkar et al. 2013). More recently, a randomized, placebo-controlled, trial involving 60 patients concluded that pregabalin administration before thoracotomy is effective in reducing postoperative pain, but in this study pregabalin did not form part of a multimodal analgesic strategy (Sattari et al. 2018).

In contrast to the studies described above, there are data to support the use of pregabalin or gabapentin as part of a multimodal analgesic strategy to improve postoperative pain and reduce opioid consumption (Mishriky et al. 2015; Tiippana et al. 2007). In a systematic review of 55 studies in surgical patients, pregabalin was associated with significant reductions, compared with placebo, in pain scores and opioid consumption 24 h after surgery; however, it was also associated with significantly higher rates of sedation, dizziness, and visual disturbances (Mishriky et al. 2015). Current guidelines for the management of postoperative pain issued by the American Society of Anesthesiology recommend the use of pregabalin and gabapentin as part of a postoperative multimodal analgesia regimen: this is considered a strong recommendation with a moderate level of evidence (Chou et al. 2016).

Recommendation 24: We suggest intraoperative intravenous administration of ketamine to reduce postoperative pain after thoracic surgery. There is no evidence about the best dose and timing of administration of ketamine.

Level of evidence: Fair

Strength of recommendation: B

A systematic review of 70 randomized controlled trials including 4701 patients found that the use of intravenous ketamine for postoperative pain management resulted in consistent reductions, compared with controls, in opioid consumption, and increases in the time to first use of analgesic (Laskowski et al. 2011). The greatest benefits were seen in patients undergoing thoracic, upper abdominal or major orthopedic surgery. Based on such evidence, US guidelines for the management of postoperative pain recommend evaluating the use of intravenous ketamine in multimodal analgesia regimens (Chou et al. 2016). However, there is currently no evidence to determine the optimal dosage of perioperative ketamine. There is evidence that a single dose of ketamine may be inadequate, and therefore some authors recommend the administration of a pre-operative bolus and intraoperative maintenance dosing (Mishriky et al. 2015; Himmelseher and Durieux 2005). One randomized controlled trial in patients undergoing major abdominal surgery has found that a reduced infusion regimen (0.015 mg/kg/h infusion following a saline bolus) and a conventional low-dose regimen (0.25 mg/kg bolus and 0.125 mg/kg/h infusion for 48 h) were comparable in analgesic efficacy, in terms of postoperative opioid consumption and rates of hyperalgesia (Bornemann-Cimenti et al. 2016). Other authors have suggested that ketamine can be administered in a series of boluses depending on the duration of the procedure (Bell et al., 2006). Ketamine should be used with caution in elderly patients.

Recommendation 25: We suggest intraoperative intravenous administration of magnesium sulfate to reduce postoperative pain after thoracic surgery.

Level of evidence: Fair

Strength of recommendation: B

Magnesium blocks *N*-methyl-d-aspartate (NMDA) receptors, which mediate central sensitization to pain and thus contribute to postoperative pain and hyperalgesia (Ko et al. 2001; Wilder-Smith et al. 1997). Hence, many trials have investigated the use of intravenous magnesium to reduce postoperative pain (Albrecht et al. 2013). In a meta-analysis of 20 randomized trials including over 1200 surgical patients, magnesium treatment was associated with significant improvements, compared with controls, in pain at rest and on movement, and with reductions in postoperative opioid consumption (De Oliveira Jr et al. 2013). A further meta-analysis of 25 trials found that perioperative magnesium administration reduced opioid consumption, and to a lesser extent pain scores, during the first 24 h after surgery (Albrecht et al. 2013). However, other studies have reported that intravenous magnesium does not reduce postoperative pain and opioid consumption (Ko et al. 2001; Wilder-Smith et al. 1997). A study in gynecological surgery patients suggests that variability in the efficacy of magnesium may be related to baseline magnesium levels: low preoperative magnesium levels were significantly (*P* < 0.001) associated with increased postoperative pain (Ulm et al. 2016). Clinical trials have consistently shown that intravenous magnesium has a favorable safety profile, even at high doses (Albrecht et al. 2013; De Oliveira Jr et al. 2013; Fawcett et al. 1999).

Recommendation 26: There is no evidence to suggest the routine use of α_2_-agonists as part of a multimodal analgesia regimen to reduce postoperative pain after thoracic surgery. There is no consensus on the best timing and schedule for administration of these drugs.

Level of evidence: Fair

Strength of recommendation: I

A meta-analysis of 30 studies involving almost 1800 surgical patients showed that both dexmedetomidine, and to a lesser extent clonidine, reduce postoperative opioid consumption and postoperative nausea, compared with controls (Blaudszun et al. 2012). However, dexmedetomidine was associated with an increased risk of postoperative bradycardia, while clonidine increased the risks of both intraoperative and postoperative hypotension, although none of these adverse events required specific interventions, and recovery times were not prolonged (Blaudszun et al. 2012). Furthermore, in a RCT involving 10,010 patients undergoing noncardiac surgery, clonidine was associated with an increased rate of important hypotension and nonfatal cardiac arrest, compared with placebo (Devereaux et al. 2014).

Dexmedetomidine is currently approved in Italy only for sedation, and thus cannot be recommended for analgesic use in Italian settings.

Recommendation 27: We suggest considering the use of intravenous steroids as part of a multimodal approach to reduce peripheral sensibilization of inflammatory-induced pain in patients undergoing thoracic surgery. Adverse effects of single doses of steroids are of trivial clinical impact.

Level of evidence: Fair

Strength of recommendation: C

A meta-analysis of 45 studies including almost 5800 patients showed that a single perioperative dose of intravenous dexamethasone resulted in significant reductions in pain scores and opioid use, and was associated with shorter stays in the post-anesthesia recovery room, compared with placebo or antiemetic treatment (Waldron et al. 2013). A further meta-analysis of 24 randomized controlled trials found that preoperative dexamethasone, at doses > 0.1 mg/kg, had a greater analgesic effect than perioperative treatment, although there was no difference in LOS between the two dosing schedules (De Oliveira Jr et al. 2011). In a randomized, placebo-controlled trial in 64 patients undergoing uterine artery embolization, administration of dexamethasone 1 h before surgery resulted in significant reductions in postoperative concentrations of cortisol and inflammatory mediators, and less pain and severe PONV, compared with placebo (Kim et al. 2016).

Although long-term glucocorticosteroid treatment is associated with significant adverse events such as hyperglycemia, increased infection risk, bleeding, and recurrence of disease in cancer patients, such events do not appear to be a concern when dexamethasone is used as part of a multimodal analgesic strategy. Studies have generally shown few serious adverse events, and no delay in wound healing, following single perioperative doses of dexamethasone in surgical patients (De Oliveira Jr et al. 2011; Holte and Kehlet 2002; Snall et al. 2013; Thoren et al. 2009).

Recommendation 28: We recommend the use of intravenous nonsteroidal anti-inflammatory drugs (NSAIDs) to reduce peripheral sensitization to inflammation-induced pain in patients undergoing thoracic surgery. Combined use of NSAIDs and paracetamol may give a further analgesic advantage.

Level of evidence: Good

Strength of recommendation: A

A meta-analysis of 17 trials evaluating the efficacy of NSAIDs in surgical patients found that these drugs were effective in reducing a composite endpoint of pain intensity scores, supplemental analgesic consumption, and time to first analgesic consumption, compared with controls (effect size 0.39, 95% CI 0.27–0.48) (Ong et al. 2005). However, although preoperative administration reduced opioid consumption and lengthened the time to first use of rescue analgesic, it reduced postoperative pain scores in only six of 12 randomized controlled trials. NSAID treatment has also been reported to reduce opioid-related adverse events such as PONV (Gan et al. 2004; Maund et al. 2011). There is evidence that the analgesic effects of NSAIDs on postoperative pain are potentiated by concomitant administration of paracetamol (Ong et al. 2005).

A number of studies have examined the efficacy and safety of ketorolac in surgical patients. A meta-analysis of 27 randomized, double-blind, trials in 2314 patients undergoing major abdominal surgery, neurosurgery, or orthopedic surgery showed that ketorolac does not increase clinically significant bleeding, compared with controls (OR 1.1, 95% CI 0.61–2.06, *P* = 0.72); however, there appeared to be a slight trend toward more bleeding with higher doses (> 30 mg) (Gobble et al. 2014). These results suggest that increases in bleeding time observed with ketorolac are not clinically relevant, and that there does not appear to be a significant risk of postoperative bleeding with ketorolac, compared with controls.

Low doses of ketorolac (10 and 15 mg) appear to be equivalent in analgesic efficacy to ketorolac 30 mg. Although no studies were identified that directly compared the analgesic efficacy of different doses of ketorolac in thoracic surgery patients, a double-blind, randomized, controlled trial in patients with moderate or severe acute pain treated in the emergency department found no significant differences in pain score reductions or adverse event profiles between patients receiving ketorolac 10 mg, 15 mg, or 30 mg (Motov et al. 2017). These findings are consistent with those of a prospective, randomized, non-inferiority trial in patients undergoing spine surgery, which found that ketorolac 30 mg was not superior to 15 mg for postoperative pain management (Duttchen et al. 2017). Based on such findings, we suggest the use of low doses of intravenous ketorolac (15 mg 2–3 times a day) for a maximum of 2 days; however, we suggest caution in using ketorolac in elderly patients (> 65 years). Ketorolac can be also administered orally (10 mg 3–4 times a day) for a maximum of 5 days.

### Locoregional techniques

Recommendation 29: We recommend the use of locoregional anesthesia for intraoperative and postoperative pain management.

Level of evidence: Poor

Strength of recommendation: A

Recommendation 30: We recommend the use of thoracic epidural analgesia in high-risk patients or in major surgical procedures where the parietal pleura (e.g., chest wall resection) is violated (i.e., thoracotomy, thoracosternotomy, chest wall resection).

Level of evidence: Fair

Strength of recommendation: A

Recommendation 31: We recommend thoracic paravertebral block for VATS, as part of a multimodal approach.

Level of evidence: Good

Strength of recommendation: A

Recommendation 32: We recommend thoracic paravertebral block in preference to thoracic epidural analgesia in patients with known or suspected coagulopathy.

Level of evidence: Fair

Strength of recommendation: A

Multiple clinical trials have shown that, in patients undergoing open thoracotomy or other major surgical procedures, thoracic epidural analgesia (TEA) is superior to intravenous opioid administration in terms of postoperative pain relief, length of hospital stay, and incidence of postoperative complications (Hazelrigg et al. 2002; Block et al. 2003; Della Rocca et al. 2002; Meierhenrich et al. 2011; Wheatley et al. 2001). However, in patients undergoing VATS procedures, less invasive procedures such as paravertebral block (TPVB) appear to be at least as effective as TEA (Kosinski et al. 2016; Steinthorsdottir et al. 2014). There is moderate-quality evidence that TEA may reduce the risk of developing persistent postoperative pain 3–18 months after thoracotomy (Weinstein et al. 2018).

Clinical trials and meta-analyses have consistently shown that TEA and TPVB are comparable in efficacy for the management of postoperative pain in thoracotomy patients (Baidya et al. 2014; Ding et al. 2014; Júnior Ade et al. 2013; Kobayashi et al. 2013; Raveglia et al. 2014; Scarfe et al. 2016; Yamauchi et al. 2017). There is also clear evidence that TPVB is associated with fewer intraoperative complications than TEA, with improved hemodynamic stability and less need for intravenous colloid therapy (Pintaric et al. 2011), probably due to unilateral segmental block. Compared with TEA, TPVB is associated with lower rates of minor postoperative complications such as urinary retention, nausea and vomiting, and hypotension (Baidya et al. 2014; Ding et al. 2014; Raveglia et al. 2014; Scarfe et al. 2016; Biswas et al. 2016; Gulbahar et al. 2010; Yeung et al. 2016), and the majority of studies have shown no significant differences in pulmonary function and pulmonary complications between the two procedures (Ding et al. 2014; Biswas et al. 2016; Blackshaw et al. 2018). Furthermore, some studies have found that epidural anesthesia may be associated with serious complications such as epidural hematoma, epidural abscess, and nerve injury: the risk of these potentially devastating complications may be reduced with TPVB, particularly in patients with known or suspected coagulopathy (Davies et al. 2006; Horlocker et al. 2018). Although data from randomized controlled trials are lacking, several studies have shown that TPVB is associated with a low risk of bleeding complications (Naja and Lönnqvist 2001; Katayama et al. 2012; Okitsu et al. 2017). In some studies, TEA has also been associated with higher rates of procedural failure, compared with TPVB (Kosinski et al. 2016; Ding et al. 2014; Gulbahar et al. 2010; Hermanides et al. 2012). There are no studies comparing the efficacy and safety of TPVB when performed by the anesthetist before the beginning of surgery, or by the surgeon under direct vision at the end of surgery.

Together, the available evidence indicates that TPVB and TEA provide comparable analgesia in thoracotomy patients, but TPVB offers advantages in terms of its technical simplicity and better safety profile. TPVB is therefore a valid alternative to TEA, particularly in patients who are not suitable for TEA.

Recommendation 33: We suggest that intercostal nerve blockade should be considered only as a second choice for analgesia after thoracic surgical procedures.

Level of evidence: Good

Strength of recommendation: C

Several studies have shown that intercostal nerve blockade is not comparable in terms of analgesia to TEA or TPVB in thoracic surgery patients (Meierhenrich et al. 2011; Joshi et al. 2008; Wurnig et al. 2002). This is at least partially due to the shorter duration of analgesia achievable with intercostal nerve blockade (Wurnig et al. 2002; Linden et al. 2014), although a recent study has shown that this can be prolonged by a combination of intravenous and perineural dexamethasone (Maher et al. 2017). As a result, we suggest that intercostal nerve blockade should be considered only as a second choice for analgesia after thoracic surgical procedures, because more effective techniques are available. Suitable alternatives include TEA and (especially for VATS) TPVB, and possibly erector spinae plane blockade and serratus anterior plane blockade (see below).

Recommendation 34: We suggest erector spinae plane block as part of a multimodal analgesia for thoracic surgery, especially for VATS.

Level of evidence: Poor

Strength of recommendation: B

Erector spinae plane blockade (ESPB) is a recently developed fascial block that allows sensory blockade over both the posterior and anterolateral thorax. It is relatively safe and simple to administer, because it is performed in a musculofascial plane away from the neuraxis, with minimal risk of serious complications (other than local anesthetic systemic toxicity) (Forero et al. 2016; Forero et al. 2017). In an initial series of seven patients with post-thoracotomy pain syndrome, who underwent ESPB as part of a multimodal analgesia strategy, all patients experienced immediate pain relief and four experienced prolonged pain relief for 2 weeks or longer (Forero et al. 2017). Randomized controlled trials are needed to confirm the effectiveness of this technique in thoracic surgery.

Recommendation 35: We suggest the use of fascial pain blocks as part of multimodal analgesia for thoracic surgery, particularly for VATS.

Level of evidence: Fair

Strength of recommendation: B

Serratus anterior plane blockade (SPB) provides good analgesia, comparable to that provided by TEA, for acute post-thoracotomy pain, while maintaining a more stable blood pressure (Khalil et al. 2017; Okmen and Okmen 2017). Like ESPB, SPB offers a less invasive approach in patients with contraindications to more invasive techniques (Park et al. 2018). A recent placebo-controlled trial has suggested that SPB reduces postoperative pain and opioid consumption during the first 24 h after VATS (Kim et al. 2018), but further studies are needed to confirm the potential of the technique in thoracic surgery (Park et al. 2018; Okmen and Okmen 2018). Nevertheless, we suggest the use of fascial plane blocks as part of multimodal analgesia for thoracic surgery, particularly for VATS patients. A recent study, involving 60 patients undergoing minimally invasive thoracic surgery, has found that ESPB provides superior quality of recovery, with lower morbidity and better pain control, compared with SPB (Finnerty et al. 2020).

Recommendation 36: We suggest considering the use of adjuvants (i.e., opioids, dexamethasone) when loco-regional anesthesia is performed, because the use of adjuvants can potentiate and prolong the effect of local anesthetics.

Level of evidence: Poor

Strength of recommendation: C

Low- to moderate-quality evidence suggests that, when used as an adjuvant to peripheral nerve blockade in upper limb surgery, both perineural and intravenous dexamethasone may prolong the duration of sensory blockade and reduce postoperative pain intensity and opioid consumption (Pehora et al. 2017). Specific evidence regarding the use of dexamethasone as an adjuvant in thoracic anesthesia is not available.

### Chest drainage

Recommendation 37: We suggest considering the use of a single large-bore chest tube instead of a double tube after thoracic surgery. Insertion of more than one chest tube may be considered in selected cases (e.g., bi-lobectomy or bleeding patients).

Level of evidence: Poor

Strength of recommendation: C

A meta-analysis of nine studies, including 918 patients undergoing pulmonary resection by VATS, found that approximately 50% of patients did not have a chest tube inserted. In these patients, postoperative pain scores and LOS were significantly reduced, compared with patients who had a chest tube inserted, with no difference in 30-day morbidity or re-intervention rates between the two groups (Li et al. 2018). These findings suggest that omitting the chest tube is safe and feasible in selected patients.

In patients in whom a chest tube is considered necessary, there is consistent evidence that the use of a single large-bore tube to remove both air and fluid is as effective as the use of double chest tubes (Filosso et al. 2017; Zhou et al. 2016). Furthermore, comparative studies and meta-analyses have shown that, compared with double chest tubes, the use of a single chest tube is associated with less pain, decreases in the amount and duration of drainage, and reduced healthcare costs (Zhou et al. 2016; Okur et al. 2009; Zhang et al. 2016).

Recommendation 38: We suggest considering the use of digital chest drainage systems to promote early mobilization of the patient.

Level of evidence: Fair

Strength of recommendation: B

External pleural suction is commonly used after lung resection to promote lung expansion and minimize the duration of air leakage (Lang et al. 2016; Leo et al. 2013). The AirINTrial, which involved 500 lung resection patients, found that the incidence of prolonged air leakage (defined as still having a chest drain in place 7 days after surgery) was not significantly different in patients in whom external suction was used, compared to those without suction (10% versus 14%, respectively, *P* = 0.2), although a trend toward significance favoring the use of external suction was seen in patients undergoing anatomical resection (9.6% versus 16.8%, *P* = 0.05) (Leo et al. 2013). However, a subsequent meta-analysis of eight randomized, controlled, trials found that, although the use of suction reduced the incidence of postoperative pneumothorax, it was associated with significant increases in LOS, duration of chest tube drainage, and air leak duration (Lang et al. 2016).

The effect of digital chest drainage systems on outcomes after pulmonary resection was studied in a trial including 103 patients who were randomized to either analog or digital drainage systems (De Waele et al. 2017). The use of digital systems had no significant effect on pleural fluid formation, but was associated with a significantly lower incidence of prolonged air leakage, compared with analog systems (3.8% versus 18%, respectively, *P* = 0.025). There was also a trend toward a shorter duration of chest tube drainage with digital systems, but this did not reach statistical significance. By contrast, an international randomized trial involving 381 lung resection patients found that, compared with traditional drainage systems, digital drainage systems were associated with a significantly shorter duration of chest tube placement, shorter hospital stays, and higher satisfaction scores (Pompili et al. 2014). We suggest using digital chest drainage systems, rather than traditional water seal devices, in order to promote early mobilization.

Recommendation 39: The routine use of drainage with suction is not recommended in the absence of complications, provided there is full re-expansion of the residual parenchyma after lung resection.

Level of evidence: Good

Strength of recommendation: D

In a prospective randomized trial involving 254 lung resection patients with full parenchymal re-expansion, suction drainage was found to be less effective than non-suction drainage in terms of time to chest tube removal (5.6 days versus 4.5 days, respectively, *P* = 0.0014) and incidence of prolonged air leakage (5.6% versus 0.7%, *P* = 0.032) (Gocyk et al. 2016). However, no-suction drainage was associated with a significantly higher incidence of asymptomatic residual air spaces, compared with suction drainage (9.4% versus 0.8%, respectively, *P* = 0.0018). Other studies have found that suction drainage does not reduce prolonged air leakage or duration of drainage in patients without complications such as large expiratory leaks (Alphonso et al. 2005; Brunelli et al. 2004; Cerfolio et al. 2001b; Coughlin et al. 2012; Marshall et al. 2002).

Recommendation 40: We suggest removing chest tubes in lung resection patients when liquid output is ≤ 5 cm^3^/kg/24 h of serous fluid.

Level of evidence: Poor

Strength of recommendation: B

In a prospective observational study in 88 patients who underwent posterolateral thoracotomy for lung resection, early removal of the chest tube resulted in an statistically significant improvement in static and dynamic pain scores, and in better functional respiratory outcome (Dokhan and Abd Elaziz 2016). The criteria for chest tube removal in this study were resolution of air leaks and fluid drainage ≤ 350 mL/day, provided that the drained fluid was macroscopically non-chylous and non-hemorrhagic.

Several authors have suggested that a cut-off of 3–5 cm^3^/kg of serous liquid is a good option because this is within the normal physiological range of daily pleural fluid filtration, and is suitable for early chest drain removal without increasing complications and re-admission rates (Brunelli et al. 2011; Mesa-Guzman et al. 2015; Miserocchi 1997). Based on this clinical evidence, we suggest chest tube removal when fluid output is ≤ 5 cm^3^/kg/24 h of serous liquid.

### ICU admission

Recommendation 41: We do not recommend systematic ICU admission after thoracic surgery.

Level of evidence: Poor

Strength of recommendation: D

Postoperative pulmonary complications occur in as many as 15–40% of patients after major thoracic surgery, and are associated with prolonged LOS, and poor long-term outcomes (Brunelli et al. 2009; Agostini et al. 2018). Although VATS procedures are associated with a reduced incidence of postoperative pulmonary complications, compared with thoracotomy, such complications still lead to significant short-term morbidity and mortality in these patients (Agostini et al. 2018). Implementation of appropriate postoperative medical strategies, and monitoring and treatment of high-risk patients in dedicated care units, are aimed at improving postoperative outcomes (Brunelli et al. 2009).

Currently, many centers routinely admit patients to the ICU after surgery, whereas in others ICU admission is reserved for patients requiring ventilator support, emergency treatment of perioperative complications, or both (Brunelli et al. 2009). Multiple preoperative factors can influence the likelihood of postoperative admission to the ICU in patients undergoing lung resection (Brunelli et al. 2009; Agostini et al. 2018; Ferguson et al. 2009; Brunelli et al. 2008; Brunelli et al. 2005; Cywinski et al. 2009; Dulu et al. 2006; Keegan et al. 2007; McCall et al. 2015; Pinheiro et al. 2015). These include open thoracotomy, rather than VATS (Brunelli et al. 2008; Dulu et al. 2006; McCall et al. 2015; Pinheiro et al. 2015), more extensive resection (Cywinski et al. 2009), and impaired preoperative lung function or pulmonary comorbidities such as chronic obstructive pulmonary disease (COPD) (Brunelli et al. 2008; Cywinski et al. 2009; Pinheiro et al. 2015). However, there is evidence that routine admission of thoracic surgery patients to the ICU does not reduce mortality rates (Brunelli et al. 2005), and may result in inappropriate ICU admission, increased healthcare costs, delayed mobilization, and increased risks of nosocomial infections (Brunelli et al. 2009). To date, no studies have compared outcomes in thoracic surgery patients admitted to ICUs, high dependency units (HDUs), or surgical wards (Brunelli et al. 2009), and there are no data to identify patients who might benefit from postoperative intensive care, or to determine the necessary degree of postoperative care for an individual patient.

For these reasons, we do not recommend systematic ICU admission after thoracic surgery. We suggest postoperative admission of high-risk patients to dedicated care units (HDUs or dedicated thoracic surgical wards). These facilities may allow ICU admission to be limited to patients requiring support for organ failure. Identification of high-risk patients, and management of their postoperative course, should be planned according to the number and type of complications, and the available resources. ERS/ESTS working group recommendations (Brunelli et al. 2009) state that lung resection patients should be managed in a dedicated thoracic surgical ward or respiratory HDU (Scala et al. 2011) if available, and that ICU admission should be limited to patients requiring organ support. The appropriateness of this policy, and its influence on early outcomes, is still controversial.

Recommendation 42: We recommend that, in adult patients undergoing thoracic surgery, oral intake, including clear liquids, can be initiated 4–6 h after surgery, in the absence of nausea and vomiting. Oral intake should, however, be adapted to individual tolerance.

Level of evidence: Fair

Strength of recommendation: A

Although it has traditionally been believed that enteral nutrition should not be resumed in postoperative surgical patients until normal bowel function has been restored, studies have consistently shown that early resumption of oral feeding is safe and well tolerated, and is associated with decreased wound morbidity, fewer septic complications, and less weight loss, compared with delayed enteral nutrition (Warren et al. 2011). Hence, early oral feeding has been endorsed in a number of guidelines in different surgical settings, including the ERAS/ESTS lung surgery guidelines (Batchelor et al. 2019; Muehling et al. 2008; Smith et al. 2011; Weimann et al. 2017; Nelson et al. 2016; Nygren et al. 2013). In patients undergoing lung resection, early resumption of oral feeding does not depend on the surgical technique (open versus minimally invasive) (Batchelor et al. 2019; Smith et al. 2011; Jones et al. 2013). Hence, we recommend that, in the absence of nausea and vomiting, oral intake, including clear liquids, can be initiated 4–6 h after surgery in adult patients undergoing elective pulmonary lobectomy. Oral intake should, however, be adapted according to the individual patient’s tolerance and the type of surgery carried out.

Recommendation 43: We recommend early mobilization of patients within the first 24 h after both minor and major thoracic surgery.

Level of evidence: Fair

Strength of recommendation: A

Recommendation 44: We recommend a physiotherapy program after thoracic surgery.

Level of evidence: Fair

Strength of recommendation: A

Delayed mobilization in patients undergoing lung resection is predictive of increased postoperative morbidity and delayed hospital discharge (Das-Neves-Pereira et al. 2009; Rogers et al. 2018), and hence early ambulation and physiotherapy have been recommended irrespective of the surgical approach (Nygren et al. 2013). Several studies have shown that ERAS programs that include early ambulation are feasible in lung resection patients, and can improve outcomes (Das-Neves-Pereira et al. 2009; Cywinski et al. 2009; Dulu et al. 2006; Keegan et al. 2007; McCall et al. 2015; Pinheiro et al. 2015; Scala et al. 2011; Warren et al. 2011; Nygren et al. 2013; Jones et al. 2013; Rogers et al. 2018; Dumans-Nizard et al. 2016; Kendall et al. 2017; Martin et al. 2018). There is evidence from a propensity score matching study in 524 patients that patients undergoing VATS lung resection require less physiotherapy than those undergoing open thoracotomy (Agostini et al. 2017).

Recommendation 45: We suggest considering daily chest radiographs only in selected cases under specific clinical indications.

Level of evidence: Good

Strength of recommendation: C

Two meta-analyses have concluded that routine chest radiographs offer no advantage over clinically indicated radiographs in cardiothoracic surgery patients (Sepehripour et al. 2012; Reeb et al. 2013). In one of these analyses, pulmonary pathology was detected in 2–40% of routine chest radiographs, compared with 79% (*P* = 0.005) of radiographs that were taken only when clinically indicated (Sepehripour et al. 2012). Furthermore, a prospective comparative study in cardiothoracic surgery patients in an ICU/post-ICU ward showed that the elimination of daily routine chest radiographs reduced the total number of radiographs per patient per day in the ICU, but had no effect on chest radiography practice in the post-ICU ward (Mets et al. 2007). There is also evidence that chest radiographs are poor predictors of postoperative complications in patients undergoing lung resection. In a retrospective chart review of 86 patients undergoing VATS lung resection, the sensitivity and specificity of chest radiographs for pulmonary complications ranged from 0–100% and 58–97%, respectively, depending on the reviewer, and there was only slight overall agreement between reviewers (Troquay et al. 2013). For these reasons, we suggest considering daily chest radiographs only in selected patients. Bedside, lung ultrasound may also be useful in some patients (Chiappetta et al. 2018; Touw et al. 2018).

Recommendation 46: We do not recommend the routine use of either continuous positive airway pressure (CPAP) or non invasive ventilation (NIV) to prevent postoperative pulmonary complications, prolonged length of stay, and mortality (both in ICU and in hospital) in patients undergoing major thoracic surgery. CPAP or NIV could be considered on a case by case basis in selected high-risk patients.

Level of evidence: Poor

Strength of recommendation: D

Postoperative pulmonary complications are the principal cause of mortality and morbidity after lung resection (Torres et al. 2019). Acute respiratory failure has been reported to occur in 2–30% of patients after lung resection (Lorut et al. 2014), and overall pulmonary complication rates have been reported to be as high as 49% (Nery et al. 2012). Because prolonged invasive mechanical ventilation has been shown to be an important risk factor for such complications, prophylactic non-invasive ventilation (NIV) has been proposed as a means of reducing this intubation-related risk (Riviere et al. 2011).

Although NIV offers the potential to improve lung function, unload respiratory muscles and reduce postoperative hypoxemia and atelectasis, randomized controlled trials have not shown consistent evidence that the addition of either NIV or continuous positive airway pressure (CPAP) to standard medical therapy offers no significant benefit (Lorut et al. 2014; Nery et al. 2012; Aguilo et al. 1997; Barbagallo et al. 2012; Danner et al. 2012; Garutti et al. 2014; Liao et al. 2010; Perrin et al. 2007). In a recent Cochrane review of eight trials involving a total of 486 patients, there were no significant differences between patients receiving NIV and control groups in terms of pulmonary complications (RR 1.03, 95% CI 0.72–1.47), intubation rates (RR 0.55, 95% CI 0.25–1.00), mortality (RR 0.60, 95% CI 0.24–1.53), length of ICU stay (mean difference − 0.75 days, 95% CI − 3.93–2.43) or length of hospital stay (mean difference − 0.12 days, 95% CI − 6.15–5.90) (Torres et al. 2019). However, the quality of the evidence was poor, due to the limited number of studies, heterogeneity of the patient populations and of the scheduled ventilator treatment, small sample sizes, and low frequencies of outcomes (Torres et al. 2019). However, it could be speculated that selected patients at higher risk of developing pulmonary complications (e.g., obese patients or patients with COPD, obese, chronic heart failure, or chronic hypersecretion) are likely to benefit from the administration of CPAP or NIV in addition to standard medical and physiotherapy, consistent with the established use of these techniques for the prevention of post-extubation failure (Rochwerg et al. 2017; Scala and Pisani 2018).

Recommendation 47: We suggest the use of NIV or CPAP to treat acute respiratory failure complicating thoracic surgery.

Level of evidence: Poor

Strength of recommendation: B

One small study (*n* = 24) in patients with acute hypoxemic respiratory insufficiency after lung resection found that the addition of NIV to standard therapy was associated with significant reductions, compared with controls, in the need for endotracheal mechanical ventilation (20.8% versus 50%, respectively, *P* = 0.035) and 120-day mortality (12.5% versus 37.5%, *P* = 0.045); however, there were no differences in length of ICU and hospital stays between the two groups (Auriant et al. 2001). On the basis of these findings, it is suggested that NIV or CPAP could be used in the management of acute respiratory insufficiency following thoracic surgery, but it should be noted that the availability of only a single study limits the strength of this recommendation.

However, it should be remembered that NIV is associated with a number of adverse events (e.g., poor compliance, leaks, sensory dysfunction, hypersecretion, unprotected airways, patient-ventilator asynchronies) that are likely to be associated with the need for intubation (Scala and Pisani 2018). Furthermore, NIV failure occurs in approximately 20% of patients, and is associated with increased rates of nosocomial pneumonia and postoperative mortality (Riviere et al. 2011). In a prospective study of 664 patients admitted to the ICU after lung resection or pulmonary thrombendarterectomy, four independent risk factors for NIV failure within the first 48 h were identified: increased respiratory rate (OR 4.17, 95% CI 1.63–10.67), increased Sequential Organ Failure Assessment (SOFA) score (OR 3.05, 95% CI 1.12–8.34), number of fiberoptic bronchoscopies performed (OR 1.60, 95% CI 1.01–2.54), and number of hours on NIV (OR 1.06, 95% CI 1.01–1.11) (Riviere et al. 2011). Risk stratification of candidates for thoracic surgery is likely to be useful for selecting sub-sets of patients who may benefit from either prophylactic or therapeutic NIV. These might include patients with COPD or severely impaired respiratory function (Danner et al. 2012; Garutti et al. 2014; Perrin et al. 2007) and obese patients (Stephan and Berard 2017). Further research is needed to clarify the potential usefulness of prophylactic or therapeutic NIV in such groups, and to determine the most efficacious scheduled regimens.

Recommendation 48: We suggest considering the use of high-flow nasal cannula oxygen therapy as an alternative or integrative support to CPAP or NIV to prevent or treat acute respiratory failure complicating thoracic surgery.

Level of evidence: Poor

Strength of recommendation: C

High-flow nasal cannula (HFNC) oxygen therapy is considered to be a non-invasive form of respiratory assistance for spontaneously breathing hypoxemic patients with early stages of acute respiratory failure. This technique delivers high inspiratory flow rates (up to 60 L/min) that match the oxygen demands of ventilated patients; in addition, HFNC oxygen therapy offers good comfort, efficient wash-out of the upper airway and clearance of CO_2_, provision of adequate humidification, and reduction of respiratory effort (although this latter effect is less than can be achieved with NIV) (Stephan and Berard 2017). A post hoc analysis of a large randomized trial in obese patients undergoing major thoracic surgery investigated the impact of HFNC on rates of treatment failure, defined as the need for re-intubation or switching to alternative treatments, or premature discontinuation (Stephan and Berard 2017). This analysis found that HFNC is not inferior to NIV in terms of treatment failure rates (13.3% versus 15.4%, respectively, *P* = 0.62), ICU mortality (2.2% versus 5.9%, *P* = 0.22), length of ICU stay (median 5.0 versus 4.0 days, *P* = 0.63), or length of hospital stay (median 10.0 versus 11.1 days, *P* = 0.71). However, skin breakdown at 24 h was significantly more common with NIV than with HFNC (9.2% versus 1.6%, respectively, *P* = 0.01).

On the basis of these findings, it is suggested that HFNC may be considered as an alternative to CPAP or NIV for the prevention or treatment of acute respiratory failure complicating thoracic surgery. It should be noted that the lack of corroborating randomized trials limits the strength of this recommendation. However, the demonstration of the effectiveness and acceptability of HFNC in milder degrees of acute (particularly hypoxemic) respiratory failure is consistent with the potential use of HFNC in patients with postoperative pulmonary complications following major thoracic surgery (Rochwerg et al. 2019). It should also be noted that the integrated use of HFNC during times off NIV could be an effective strategy, especially in patients showing poor tolerance to the NIV interface (Scala and Pisani 2018; Longhini et al. 2019).

Recommendation 49: For prophylaxis and management of atrial fibrillation after thoracic surgery, we recommend reference to the Society of Thoracic Surgery (STS) 2011 Guidelines.

Level of evidence: Good

Strength of recommendation: A

Postoperative cardiac arrhythmias, particularly atrial fibrillation, occur in approximately 10–20% of patients undergoing major noncardiac thoracic surgery, including both thoracotomy and VATS lobectomy (Garner et al. 2017; Onaitis et al. 2010; Park et al. 2007). Potential risk factors for atrial fibrillation include increasing age, male gender, hypertension, comorbidities such as COPD or heart failure, extent of lung resection, and postoperative infection (Batchelor et al. 2019; Garner et al. 2017; Onaitis et al. 2010). Postoperative atrial fibrillation can lead to hemodynamic instability, potentially prolonging ICU and hospital stay (Frendl et al. 2014). Furthermore, atrial fibrillation may persist beyond hospital discharge in a proportion of patients, and some patients may require long-term anticoagulation (Garner et al. 2017).

It is recommended that the Society of Thoracic Surgery (STS) 2011 guidelines for the prophylaxis and management of atrial fibrillation (Fernando et al. 2011) should be followed in patients undergoing pulmonary lobectomy. These guidelines recommend pharmacological prophylaxis with β-blockers or diltiazem: amiodarone is not recommended for patients undergoing pneumonectomy. Electrical cardioversion is recommended for patients who develop hemodynamically unstable atrial fibrillation, and for patients with symptomatically intolerable atrial fibrillation in whom treatment with metoprolol (or diltiazem for patients with severe COPD), alone or followed by flecainide, is ineffective. Anticoagulation with aspirin (for patients at low thromboembolic risk), or warfarin or heparin (for high-risk patients), is recommended for patients with persistent or recurrent atrial fibrillation after 24 h of metoprolol treatment (Fernando et al. 2011). It should be noted, however, that to date no scoring system has been developed to identify lung resection patients at high risk of atrial fibrillation, although promising results have been obtained with the CHADS_2_ score (Kotova et al. 2017). Furthermore, there is little evidence that prophylaxis for atrial fibrillation improves outcomes after thoracic surgery [1].

## Conclusions

Anesthesia in patients undergoing thoracic surgery is a complex undertaking that requires a multidisciplinary approach to risk assessment, perioperative monitoring, and postoperative care. Recognizing this, the PACTS group has sought to identify critical issues in the preoperative, intraoperative and postoperative care of patients undergoing lung resection, and to provide appropriate guidance. Wherever possible, our recommendations are based on good-quality supporting evidence: where such evidence is limited, the recommendations are framed as suggestions or possibilities for consideration. In a few cases, there was insufficient evidence to make formal recommendations: in such cases, our guidance is based on expert opinion, supported by published literature where possible.

Our literature reviews and discussions highlighted the importance of the choice of anesthetic and lung isolation procedure, attention to airway management, and comprehensive monitoring of vital signs, hemodynamics, neuromuscular blockade, and depth of anesthesia, for achieving optimal outcomes. Postoperatively, a multi-modal analgesic strategy that includes pre-emptive analgesia and locoregional blockade is required for optimal pain control. Finally, decisions on ICU care, chest drainage, and other interventions should be individualized for each patient.

The ERAS lung surgery guidelines (Batchelor et al. 2019) were published while our recommendations were in development. We believe that these recommendations extend and complement those of the ERAS guidelines for a number of reasons. First, aspects of anesthesiologic care such as depth of anesthesia monitoring, neuromuscular blockade, and hemodynamic monitoring are covered in greater detail than in the ERAS guidelines. In addition, our recommendations focus specifically on elective surgery for lung cancer.

It is hoped that these recommendations will help to achieve optimal postoperative outcomes in the greatest number of thoracic surgery patients. Further refinement of our recommendations can be anticipated as the literature continues to evolve.

## Data Availability

Data sharing is not applicable to this article as no datasets were generated or analyzed during the current study.

## Abbreviations

AIPO: Italian Association of Hospital Pulmonologists (Associazione Italiana Pneuomologi Ospedalieri)
AKI: Acute kidney injury
BIS: Bispectral index
CI: Confidence interval
COPD: Chronic obstructive pulmonary disease
CPAP: Continuous positive airway pressure
DLT: Double-lumen tube
ERAS: Enhanced recovery after surgery
ERS: European Respiratory Society
ESA: European Society of Anaesthesiologists
ESPB: Erector spinae plane blockade
ESTS: European Society of Thoracic Surgeons
FiO_2_: Inspired oxygen fraction
HDU: High dependency unit
HFNC: High-flow nasal cannula
HR: Hazard ratio
ICU: Intensive care unit
LOS: Length of stay
MINS: Myocardial injury after non-cardiac surgery
NIV: Non-invasive ventilation
NMBA: Neuromuscular blocking agent(s)
NMDA: *N*-methyl-d-aspartate
NSAID: Nonsteroidal anti-inflammatory drug
OR: Odds ratio
PACTS: Perioperative Anesthesia Care in Thoracic Surgery
pEEG: Processed electroencephalography
PEEP: Positive end-expiratory pressure
PICO: Patients, intervention, comparison, outcome
POCD: Postoperative delirium and cognitive dysfunction
PONV: Postoperative nausea and vomiting
RCT: Randomized controlled trial
RR: Risk ratio
SIAARTI: Italian Society of Anesthesia, Analgesia, Resuscitation, and Intensive Care (Società Italiana di Anestesia Analgesia Rianimazione e Terapia Intensiva)
SIC: Italian Society of Surgery (Società Italiana di Chirurgia)
SICT: Italian Society of Thoracic Surgery (Società Italiana di Chirurgia Toracica)
SIET: Italian Society of Thoracic Endoscopy (Società Italiana di Endoscopia Toracica)
SIP/IRS: Italian Society of Pneumology (Società Italiana di Pneumologia)
SOFA: Sequential Organ Failure Assessment
SPB: Serratus anterior plane blockade
STS: Society of Thoracic Surgery
TEA: Thoracic epidural analgesia
TPVB: Thoracic paravertebral block
USPSTF: United States Preventive Services Task Force
VATS: Video-assisted thoracic surgery
